# Frequency, distribution and immunologic nature of infusion reactions in subjects receiving pegloticase for chronic refractory gout

**DOI:** 10.1186/s13075-017-1396-8

**Published:** 2017-08-17

**Authors:** Leonard H. Calabrese, Arthur Kavanaugh, Anthony E. Yeo, Peter E. Lipsky

**Affiliations:** 1 Cleveland Clinic, Department of Rheumatic & Immunologic Diseases, Cleveland, OH 44195 USA; 2 University of California San Diego, Division of Rheumatology, Allergy and Immunology, La Jolla, CA 92093 USA; 3 Horizon Pharma, Lake Forest, IL 60045 USA; 4 AMPEL BioSolutions, LLC, 250 W. Main Street, Charlottesville, VA 22902 USA

**Keywords:** Anaphylaxis, Gout, Hypersensitivity, Pegloticase

## Abstract

**Background:**

To assess frequency and distribution of infusion reactions (IRs) in responders and nonresponders in randomized clinical trials (RCTs) of intravenous pegloticase and the utility of the National Institute of Allergy and Infectious Disease/Food and Allergy and Anaphylaxis Network (NIAID/FAAN) criteria for identifying anaphylaxis in subjects experiencing IRs.

**Methods:**

IRs from two RCTs of pegloticase were evaluated and categorized as anaphylaxis, hypersensitivity, or other. Serum levels of tryptase and total hemolytic complement (CH50) were evaluated at the time of all IRs. Frequency of IRs by each category was evaluated in all subjects, responders or nonresponders to pegloticase.

**Results:**

There were 113 IRs in 1695 infusions. Of the 113 IRs, 6 met criteria for anaphylaxis, 53 had one feature of anaphylaxis and were designated as “hypersensitivity”, and 54 had no features and were designated “other”. In subjects receiving pegloticase every 2 weeks (Q2w), a total of 852 infusions were administered and the IR frequency was 0.5% in responders and 9.7% in nonresponders. In subjects receiving pegloticase every 4 weeks (Q4w), a total of 846 infusions were given and the IR frequency was 2.6% in responders and 12.2% in nonresponders. There were no differences among the three categories of IRs with regard to clinical course or biochemical evidence of immune activation determined by CH50 or tryptase levels.

**Conclusion:**

IRs mostly occurred in nonresponders. NIAID/FAAN criteria for anaphylaxis did not identify pegloticase-related IRs as having a higher frequency of immune activation or a more severe course. The results are consistent with the conclusion that discontinuance of pegloticase if uric acid rises to >6 mg/dL will decrease the frequency of IRs.

## Background

The uricolytic pegloticase is a mammalian recombinant uricase conjugated to monomethoxypoly (ethylene glycol) [1]. It is an enzymatic alternative to conventional urate-lowering agents that is indicated for the treatment of adult patients with gout refractory to conventional therapy or for whom these drugs are contraindicated [2]. The efficacy and safety of pegloticase was demonstrated in two replicate 6-month, randomized placebo-controlled trials (RCTs) that enrolled patients with symptomatic gout and plasma uric acid concentrations ≥8 mg/dL who had failed to respond to or were intolerant of allopurinol [3]. The most common adverse events in these trials, other than gout flares, were infusion reactions (IRs), which were defined as any infusion-related adverse event or cluster of temporally related events that occurred during or within 2 hours after drug infusion that could not be reasonably attributed to another cause [4]. Results from this trial indicated that 49 of 85 (58%) of pegloticase-treated patients in this study were classified as nonresponders, but continued to receive pegloticase. Assessment of immunogenicity indicated that development of high-titer anti-drug antibodies occurred significantly more frequently in nonresponders vs. responders and were associated with the occurrence of IRs [3, 5], but a full analysis of all IRs and their relationship to responder/nonresponder status has not been carried out.

Some of the subjects with IRs had clinical features suggestive of anaphylaxis. There was no pre-specified definition of anaphylaxis in the pegloticase study protocols, and there were no reports of anaphylaxis in the database from investigators who participated in the RCTs [4]. However, a post-hoc analysis carried out by the United States Food and Drug Administration (FDA) identified 14 cases of “anaphylaxis” or potential anaphylaxis among the 273 patients tested in the phase 2 and 3 clinical development programs for pegloticase [4]. An additional analysis using the National Institute of Allergy and Infectious Disease (NIAID)/Food Allergy and Anaphylaxis Network (FAAN) criteria for anaphylaxis [6] indicated that 3 of 85 patients treated with pegloticase every 2 weeks (Q2w) had IRs meeting these criteria [4]. The reason for the discrepancy remains unclear.

The NIAID/FAAN criteria for anaphylaxis were not developed as a diagnostic tool, but they are nevertheless widely used for this purpose. Importantly, studies aimed at validating them have produced mixed results, noting high sensitivity, but low specificity [7, 8]. The limitations of the NIAID/FAAN criteria have also been noted in the 2015 practice parameter on anaphylaxis from the American Academy of Allergy, Asthma and Immunology; the American College of Allergy, Asthma and Immunology; and the Joint Council of Allergy, Asthma and Immunology [9].

The objectives of the present study were to re-evaluate all IRs from the two pegloticase RCTs according to the NIAID/FAAN criteria, to relate their occurrence to the responder status of the subject as a surrogate for the presence of significant titers of anti-drug antibodies, and to determine whether or not they were associated with a more severe clinical course and/or evidence of greater immune system activation.

## Methods

Information about IRs was obtained from the data collected from the replicate, 6-month RCTs of pegloticase (studies CO405 and CO406 or GOUT 1 and 2; identifier: NCT00325195), the results of which were submitted to the FDA in support of pegloticase’s approval in 2010. The two replicate, randomized, double-blind, placebo-controlled trials (CO405 and CO406) were conducted between June 2006 and October 2007 at 56 rheumatology practices in in the USA, Canada, and Mexico. The studies received Institutional Review Board (IRB) approval at each site as previously disclosed [3]. Written informed consent and Health Insurance Portability and Accountability Act assurances were completed for each participant before enrollment and permitted post hoc analysis. The authors’ IRBs did not require further approval for these analyses.

The design of these two studies has been previously described [3]. An important feature of these two trials was that pegloticase treatment was continued in patients who lost their plasma uric acid lowering response to pegloticase [3]. Loss of this response has been demonstrated to be associated with the development of antibodies to the polyethylene glycol (PEG) portion of the pegloticase molecule and increased risk for the development of IRs [3, 5].

A total of 225 patients were randomized, and 212 received one or more infusions of study treatment in the RCTs [3]. All patients received IR prophylaxis prior to each infusion in the RCTs as follows: the non-sedating H_1_-antihistamine fexofenadine, 60 mg on the night preceding and the morning of infusion; acetaminophen, 1000 mg the morning of infusion; and hydrocortisone, 200 mg intravenous (IV) given immediately before the infusion [3, 4]. A responder was defined as a patient with plasma uric acid <6.0 mg/dL for ≥80% of the time during both months 3 and 6, the periods extending respectively from the week-9 infusion to just prior to the week-13 infusion, and from the week-21 infusion to the week-25 final study visit. A total of 113 IRs were encountered during the RCTs. An independent assessor reviewed each patient record with no knowledge of responder status and IRs were classified as anaphylaxis if they met the first set of NIAID/FAAN criteria (Table 1). IRs meeting only one of the NIAID/FAAN criteria were categorized as “hypersensitivity”, and those meeting none of the criteria were categorized as “other”.

**Table 1:** NIAID/FAAN criteria for the occurrence of anaphylaxis - category 1 [6]

Anaphylaxis is likely when any one of these three criteria is fulfilled:	
1. Acute onset of illness (minutes to several hours) with involvement of the skin, mucosal tissue, or both (e.g., generalized hives, pruritus or flushing, swollen lips, tongue, or uvula) and at least one of the following: • Respiratory compromise (e.g., dyspnea, wheeze or bronchospasm, stridor, reduced peak expiratory flow, hypoxemia) • Reduced blood pressure or associated symptoms of end-organ dysfunction (e.g., hypotonia (collapse), syncope, incontinence)	

*NIAID* National Institute of Allergy and Infectious Disease, *FAAN* Food and Allergy and Anaphylaxis Network

To determine whether immunologic mechanisms were involved in IRs, the relationship between these events and total hemolytic complement (CH50) decreases or increases in serum tryptase levels (a measure of mast cell degranulation) were evaluated. Samples for tryptase were taken at the times of IRs and serum CH50 levels were obtained routinely during the RCTs to determine whether anti-pegloticase antibodies resulted in the formation of immune complexes with resultant complement decreases. Samples for the determination of CH50 and antibody titers were obtained at the same time points. Tryptase and CH50 levels were determined immediately. A decrease in complement was defined as a value below the lower bound of the 95% confidence interval for all samples analyzed from subjects receiving placebo infusions (*n* = 330). Data on CH50 was available from 97/113 IRs and results from tryptase determinations were available on 100/113 IRs.

## Results

**Fig. 1:**
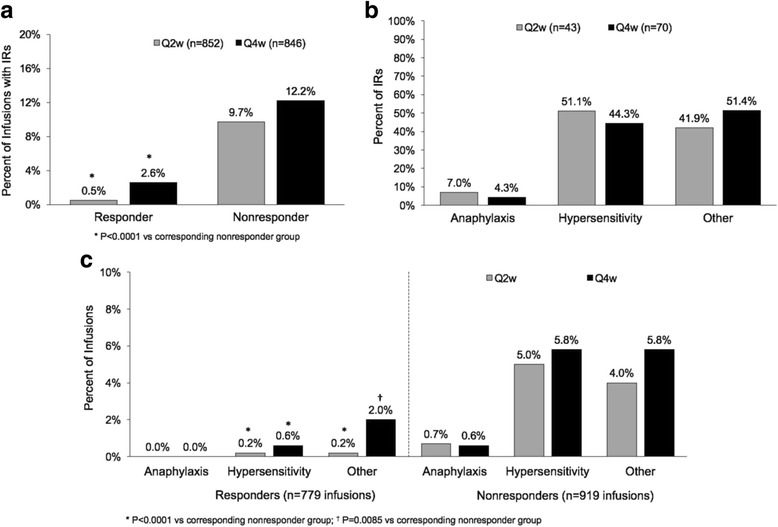
**a** Infusion reactions (*IRs*) in responders and nonresponders in the groups receiving pegloticase every 2 weeks (*Q2w*) and every 4 weeks (*Q4w*). **b** IRs classified as anaphylaxis, hypersensitivity, or other in the Q2w and Q4w pegloticase dosing groups. **c** IRs classified as anaphylaxis, hypersensitivity, or other for responders and nonresponders in the Q2w and Q4w pegloticase dosing groups

Results from the primary publication of the two pivotal clinical trials for pegloticase indicated that IRs occurred in 25.9% of patients treated with pegloticase biweekly, 40.5% of those treated with pegloticase monthly, and 4.6% of those who received placebo. Overall, 113 IRs occurred in 1695 infusions. Of a total of 852 infusions in subjects receiving pegloticase Q2w, 43 (5.0%) were associated with IRs as were 70 (8.3%) of 843 infusions in subjects receiving pegloticase Q4w. IRs occurred significantly more often in patients who received pegloticase Q4w vs. those treated Q2w (*P* = 0.04) and much more often in patients who lost their plasma urate lowering response to pegloticase (nonresponders) versus those who maintained their response (responders) (*P* < 0.0001) (Fig. 1a).

Overall, 6 (5.3%) of the 113 IRs met the NIAID/FAAN criteria for anaphylaxis (Table 2). No demographic features of the subjects distinguished those who had IRs meeting the criteria for anaphylaxis. The IRs that met the criteria for anaphylaxis occurred during the first through fifth infusions, with manifestations occurring between 13 and 135 minutes after the onset of the infusion. Of the five subjects who had determinations of serum urate at the time of the IR, all five had levels >6.0 mg/dL. IRs meeting the criteria for anaphylaxis were characterized by the investigator as mild to moderate and none was described as anaphylaxis. None of these IRs was fatal, none required advanced support, and three of the six subjects continued to receive pegloticase infusions. Of these three subjects, one completed the trial with no further IRs, one subsequently expired from an unrelated cardiac arrest, but had no further IRs and one completed the RCT with no further IRs, but developed IRs after about 6 months of the open-label extension, but these had no characteristics of anaphylaxis.

**Table 2:** Infusion reactions meeting criteria of anaphylaxis

Patient	Sex	Race	Weight (kg)	Tophi present	Regimen	Responder status	SUA at time of IR (mg/dL)	Dose of pegloticase when IR occurred	Time from infusion initiation to IR (min)	Severity of the adverse event	Signs and symptoms	Action taken	Disposition
1	Male	White	108.9	No	8 mg Q2w	Nonresponder	9.1	5	13	Moderate	Dyspnea, tongue edema	Stop infusion	Exit trial
2	Male	White	107.9	Yes	8 mg Q2w	Nonresponder	6.9	3	135	Mild	Urticaria, wheezing	Stop infusion	Continue
3	Male	Native Hawaiian or Pacific islander	98.0	Yes	8 mg Q2w	Nonresponder	9.2	1	18	Mild	Flushing, hypotension, tachycardia, urticaria	Stop infusion	Exit trial
4	Male	Black or African American	103.3	Yes	8 mg Q4w	Nonresponder	9.2	3	33	Moderate	Dyspnea, hypoxia, tachycardia, urticaria	Stop infusion; treatment with diphenhydramine and salbutamol	Exit trial
5	Male	White	111.1	No	8 mg Q4w	Nonresponder	9.8	2	15	Moderate	Cough, dyspnea, flushing	Stop infusion	Continue complete trial
6	Male	White	53.1	Yes	8 mg Q4w	Nonresponder	NA	2	5	Moderate	Dyspnea, erythema, pruritus	Interrupted infusion, but completed; treatment with diphenhydramine	Continue

*IR* infusion reaction, *NA* not available, *Q2w* every 2 weeks, *Q4w* every 4 weeks, *SUA* serum uric acid

Fifty-three (46.9%) IRs had only one feature and were classified as hypersensitivity and 54 (47.8%) had no features and were classified as other. Of the 70 IRs associated with Q4w administration, 3 (4.3%) met the criteria for anaphylaxis, 31 (44.3%) for hypersensitivity, and 36 (51.4%) were categorized as other (Fig. 1b). Of the 43 IRs in subjects receiving pegloticase Q2w, 3 (7.0%) were categorized as anaphylaxis, 22 (51.1%) as hypersensitivity, and 18 (41.9%) as other (Fig. 1b). Each class of IR occurred significantly more often in nonresponders compared to responders (Fig. 1c). None of the IRs categorized as anaphylaxis occurred in responders.

Complement levels were obtained before each infusion. It is notable that the frequency of decreased CH50 levels was significantly (*P* < 0.0001) greater at the time of an IR (21/97 samples, 21.6%) compared to the frequency of decreased CH50 levels in subjects receiving pegloticase but not experiencing an IR (58/1036 samples, 5.6%). In contrast, the frequency of samples with decreased CH50 levels was not different between subjects receiving pegloticase and not experiencing an IR and subjects receiving placebo (16/330, 5.0%, *P* = 0.72).

**Fig. 2:**
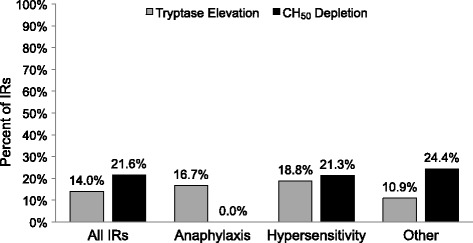
Tryptase elevation and total hemolytic complement (*CH50*) depletion in all infusion reactions (*IRs*) and those classified as anaphylaxis, hypersensitivity, or other. There was no significant difference in the distribution of tryptase elevation or complement depletion in the various groups (*P* = 0.56, chi^2^ test)

Tryptase levels were obtained at the time of an IR. Similar to the results on CH50 analysis, only a minority of IRs were associated with an elevation in tryptase levels (14.0%). Assessment of complement decreases and tryptase elevation did not differentiate between IRs categorized as anaphylaxis, hypersensitivity, or other (Fig. 2). The distribution of immunologic abnormalities was not significantly different in the three IR categories, with 16.7%, 18.8%, and 10.9% of anaphylaxis, hypersensitivity, and other IRs, respectively, having increased tryptase; and 0%, 21.3%, and 24.4%, respectively, having decreased complement. Differences in the distribution of these abnormalities in the various IR categories were not statistically significant (*P* = 0.56, chi^2^ test).

## Discussion

This post-hoc evaluation of IRs in patients with chronic refractory gout treated with pegloticase indicated 113 of these events over the 1695 pegloticase infusions carried out during the RCTs. These events occurred significantly more often in nonresponders. Importantly, all of the IRs noted resolved rapidly and completely with supportive measures and no patient required intubation, mechanical ventilatory support, pressor support, or hospitalization [3, 10]; 60.7% of patients who experienced IRs continued to receive pegloticase. Three of the six patients with IRs that met the criteria for anaphylaxis remained in the study and continued to receive pegloticase.

Categorization indicated that six of the IRs (all in nonresponders) met the NIAID/FAAN criteria for anaphylaxis. However, these events could not be differentiated from other IRs classified as hypersensitivity or other, on the basis of either clinical course or biochemical parameters. This suggests that these criteria do not have high specificity for distinguishing anaphylaxis, as defined by severe clinical signs and symptoms and biochemical evidence of immune activation, from other events. This result is consistent with the observation that the NIAID/FAAN criteria have a positive predictive value (PPV) of 69% meaning that of 100 people identified as being highly likely to have anaphylaxis, only 69 would have anaphylaxis, and 31 would not [7, 11]. A more recent evaluation of the NIAID/FAAN criteria indicated a slightly lower PPV of 63.7% [8]. Given these results, it is perhaps not surprising that identification of a subject as having anaphylaxis according to the NIAID/FAAN criteria did not predict biochemical evidence of immune activation or increased clinical severity of the event compared to other IRs. Consideration of these results requires acknowledgement of the limitations of the biochemical markers employed for assessment of immune system activation. Increased tryptase levels are highly suggestive of an immunologically mediated reaction associated with mast cell degranulation, but may also occur following direct mast cell activation [12]. Results from several studies have also indicated that elevated tryptase levels have low sensitivity for diagnosis of anaphylaxis [13, 14]. In addition, measurement of CH50 is a functional assay associated with rather extensive complement consumption [15, 16]. Direct measurement of C4 rather than CH50 may be more sensitive to more subtle classic pathway activation of the complement system by immune complexes. Finally, there may be other mechanisms of immune activation, such as an effect on basophils not detected by these measures.

## Conclusion

The present analysis underscores an important point about IRs that has been made in prior studies [3–5]. These events occurred more often in nonresponders to pegloticase and rarely in responders. Since the development of nonresponder status largely reflected the development of anti-drug antibodies, the results suggest that anti-pegloticase antibodies contribute to the tendency to develop IRs. However, whatever the mechanism, it is not reflected in increased tryptase levels or decreased CH50. In a post-hoc analysis described in the original report of the RCTs and a follow-up study, it was noted that a loss of plasma uric acid-lowering efficacy, as reflected by a uric acid level >6 mg/dL, preceded a patient’s first IR whenever it occurred, in 87.5% of patients (91% of 22 patients treated Q2w who had IRs and 85% of 34 patients treated Q4w and had IRs) [5]. All of these results suggest that following the guidance to stop pegloticase when serum uric acid levels increase to >6 mg/dL before infusion [2], has the potential to greatly decrease the occurrence of IRs in gout patients receiving pegloticase treatment. They also indicate that in pegloticase-treated patients, the NIAID/FAAN anaphylaxis criteria did not accurately identify subjects with IRs associated with biochemical evidence of immune activation or a more severe clinical course.

## Abbreviations

CH50: Total hemolytic complement
FAAN: Food and Allergy and Anaphylaxis Network
FDA: Food and Drug Administration
IR: Infusion reaction
IRB: Institutional Review Board
IV: Intravenous
NIAID: National Institute of Allergy and Infectious Disease
PEG: Polyethylene glycol
PPV: Positive predictive value
Q2w: Every two weeks
Q4w: Every four weeks
RCT: Randomized clinical trial
